# TIPE1 function as a prognosis predictor and negative regulator of lung cancer

**DOI:** 10.18632/oncotarget.19655

**Published:** 2017-07-28

**Authors:** Xiaocheng Wu, Yunmiao Ma, Ji Cheng, Xia Li, Hui Zheng, Li Jiang, Renjie Zhou

**Affiliations:** ^1^ Department of Emergency, Xinqiao Hospital, Third Military Medical University, Chongqing 400037, China; ^2^ Departmnet of Orthopedics, Zhuji People’s Hospital, Zhejiang 311800, China

**Keywords:** TIPE1, lung cancer, prognosis, apoptosis

## Abstract

TIPE1 (tumor necrosis factor-α-induced protein 8-like 1 or TNFAIP8L1) belongs to the TIPE (TNFAIP8) family, which act as a regulator of cell death. However, the expression and biologic functions of TIPE1 in lung cancer are largely unknown. Here, we investigated the roles of TIPE1 in lung cancer. Evaluated by qRT-PCR and immunohistochemical staining, lower TIPE1 mRNA and protein expression was found in the lung tumor tissue, compared with adjacent non-tumor tissues, which positively correlated with tumor patient survival. Overexpression of TIPE1 by lentivirus system in TIPE1-downregulated lung cancer cells significantly diminished cell growth and colony formation, companied with proliferation inhibition, apoptosis induction and invasion inhibition. It was identified to be due to TIPE1-regulated Cyclin D1, Cyclin B1, caspase 8, Caspase3, MM2 and MMP9 expression. Consistently, using a homograft tumor model in Balb/c mice, we discovered that TIPE1 prevented the growth and tumor weight of murine lung cancer homografts. Our findings revealed the anti-tumor role of TIPE1 in lung cancer cells and TIPE1 might be a novel prognostic indicator for lung cancer patients.

## INTRODUCTION

Lung cancer is the leading cause of cancer deaths in both women and men, contributing to years of life lost because of premature mortality, while approximately 85% of the patients with lung cancer die of the disease within 5 years [1]. Typical risk factors for lung cancer include smoking and exposure to arsenic, chromium, radon, or air pollution [2]. Smoking is the major risk factor for lung cancer, particularly squamous cell carcinoma (SqCC) [3]. The past decade has seen the emergence of histology (squamous cell versus non-squamous) as an important determinant of therapy in non-small-cell lung carcinomas (NSCLC) [4]. However, a significant proportion of patients have tumors with therapeutically targetable molecular characteristics (mutations, fusion genes etc.), and currently, the vast majority of these actionable molecular abnormalities occur in pulmonary adenocarcinomas [5, 6]. Thus, a better understanding of the mechanisms underlying lung cancer development and progression is direly needed to design novel effective therapies for this deadliest cancer.

Tumor necrosis factor (TNF)-α-induced protein 8 (TNFAIP8) family is a newly identified group of proteins consisting of TNFAIP8 (TIPE), TIPE1 (TNFAIP8L1, TNF-α-induced protein 8-like 1), TIPE2 (TNFAIP8L2), and TIPE3 (TNFAIP8L3). All the members of TNFAIP8 family share homologous sequence at the high degrees and they are involved in regulating cell apoptosis and cell proliferation [7, 8]. But notably, apart from limited homology within the death effector domain (DED), they do not share significant sequence homology with other proteins, so TNFAIP8 family is normally considered as a novel subfamily of DED containing proteins. Previous study have demonstrated that TNFAIP8 is a negative regulator of apoptosis in certain cell types [9]. TIPE2 functioned as a regulator to be involved in the regulation of immune homeostasis, via binding to a cofactor [10]. TIPE3 was involved in phosphoinositide second messengers transporting and increased in human cancer cells [11].

Until now, only one study have shown that TIPE1 could induced hepatocellular carcinoma (HCC) cell apoptosis by targeting Rac1 [12]. But, the function and predicting role of TIPE1 in lung cancer is still confused. In the present study, we attempted to investigate the expression and predicting role of TIPE1 by lung cancer tumor microarray and found lower TIPE1 expression in the lung tumor tissue, and TIPE1 expression positively correlated with tumor patient survival. Further function research indicated that TIPE1 significantly diminished cell growth and colony formation, companied with proliferation inhibition, apoptosis induction and invasion inhibition *in vitro* and *in vivo*. Our findings revealed the anti-tumor role of TIPE1 in lung cancer cells and TIPE1 might be a novel prognostic indicator for lung cancer patients.

## RESULTS

### TIPE1 deficiency promotes lung cancer progression and correlates with a worse patient prognosis

**Figure 1:**
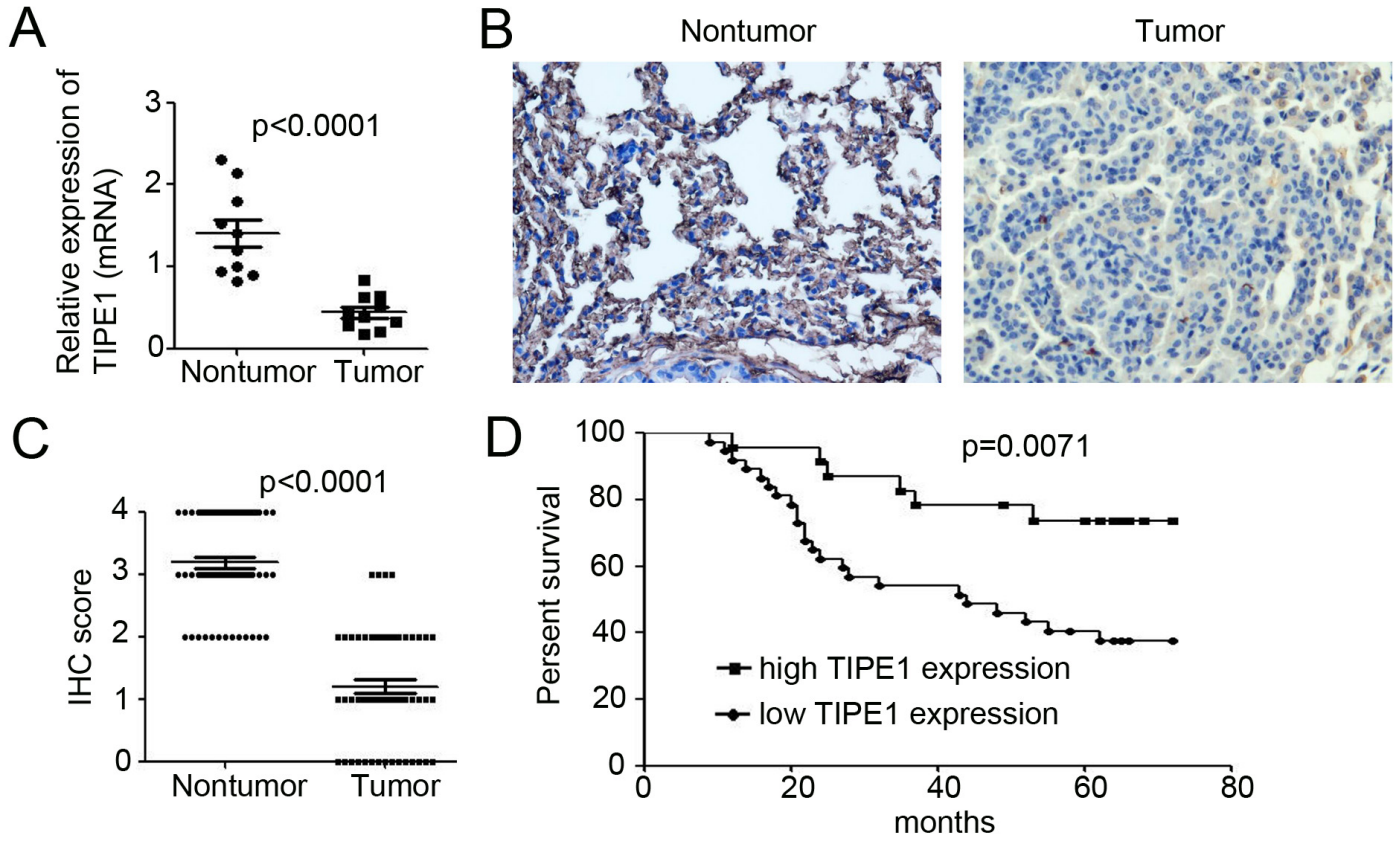
TIPE1 deficiency promotes lung cancer progression and correlates with a worse patient prognosis **(A)** qPCR analysis of TIPE1 mRNA expression in ten pairs of lung nontumor and tumor tissues. **(B)** Immunohistochemical staining of TIPE1 expression in lung tissue array, containing 60 pairs of nontumor and tumor tissues. Scale bar=10μm. **(C)** IHC score analysis of TIPE1 expression in lung nontumor and tumor tissues (n=60; p<0.0001). **(D)** Kaplan–Meier survival curve of patients with lung cancer with low expression (n=42) and high expression (n=18) of TIPE1 protein. The P value was calculated using the two-sided log-rank test.

To confirm TIPE1 expression in lung cancer tissues, we examined TIPE1 mRNA expression in ten paired nontumor and tumor tissue samples derived from lung cancer patient. qPCR results indicated that TIPE1 mRNA was 8.5-fold lower in tumor tissues than in nontumor tissues (Figure 1A). Immunohistochemistry for TIPE1 expression in lung cancer tissues array (Table 1) indicated that TIPE1-positive cells were rarely observed among lung cancer cells (Figure 1B). Meanwhile, strong and intermediate immunostaining was observed in the nontumor lung tissues (Figure 1B). In addition, mean IHC score in tumor tissues was 1.20±0.16, whereas 3.18±0.10 in nontumor tissue (n=45, p<0.0001, Figure 1C). The Kaplan–Meier survival curve demonstrated that patients with low TIPE1 expression have dramatically shorter survival than those with high TIPE1 expression (p=0.0071, Figure 1D).

**Table 1:** Analysis of the correlation between TIPE1 expression and clinic pathologic characteristics

Characteristics	Number of patients	TIPE1 expression		P value
		Low	High	
Gender(%)				**>0.05**
Male	39 (65.0)	17 (43.5)	22 (56.5)	
Female	21 (35.0)	10 (47.6)	11 (52.4)	
Age(%)				**>0.05**
≤60	28 (46.7)	12 (42.9)	16 (57.1)	
>60	32 (53.3)	15 (46.9)	17 (53.1)	
TNM stage (%)				**< 0.001**
I/II	26 (43.3)	9 (34.6)	17 (65.4)	
>II	34 (56.7)	23 (67.6)	11 (32.4)	
Histological grade(%)				**<0.001**
I/II	31 (51.7)	11 (35.5)	20 (64.5)	
>II	29 (48.3)	20 (69.0)	9 (31.0)	

### TIPE1 inhibits lung cancer growth and colony formation

**Figure 2:**
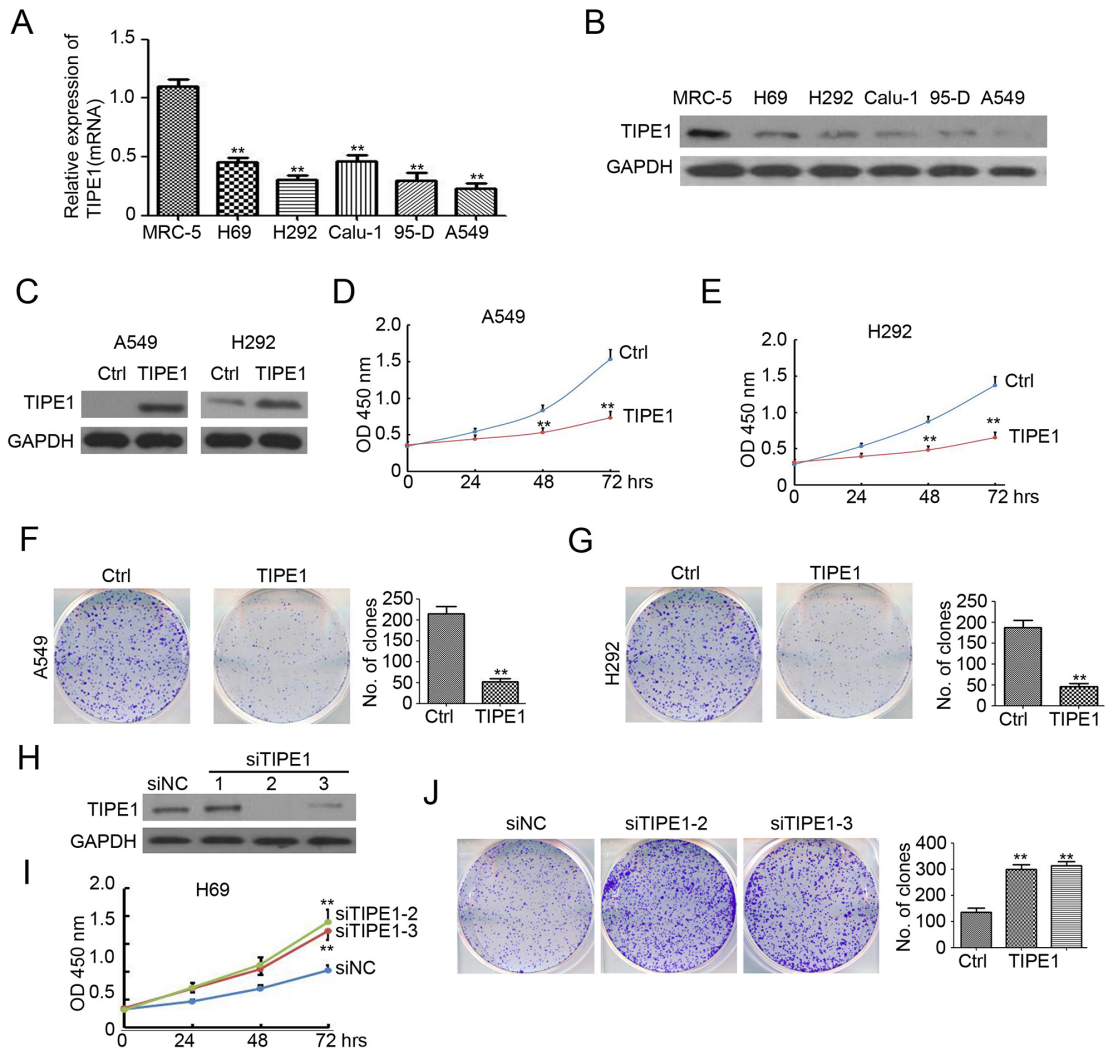
TIPE1 inhibits lung cancer growth and colony formation **(A** and **B)** TIPE1 mRNA and protein expression in five lung cancer cell lines and normal lung epithelial cells MRC-5 was determined by qPCR and western blotting, respectively. GAPDH was used as loading control. **(C)** TIPE1 protein expression in A549 and H292 cells was determined by western blotting. GAPDH was used as loading control. **(D** and **E)** Growth of A549 and H292 cells was determined by CCK8 assay (n=5; **, p<0.01, compared with Ctrl group). **(F** and **G)** Colony formation was performed in A549 and H292 cells (n=3; **, p<0.01, compared with Ctrl group).

To determine the functional effects of TIPE1 on the biological behaviors of lung cancer cells, we first detected basal TIPE1 mRNA and protein expression in five colorectal cancer cell lines and normal lung epithelial cell MRC-5 by qPCR and western blotting analysis, respectively. As shown in Figure 2A and 2B, TIPE1 expression was significantly decreased, even to undetectable levels. Thus, we employed lentivirus-based TIPE1 expressing system to infect A549 and H292 cells (20 pfu/cell). And puromycin was added to select the stable expression cells. Western blotting analysis indicated the higher expression of TIPE1 protein in TIPE1-infected A549 and H292 cells (Figure 2C). Overexpression of TIPE1 dramatically arrested cell growth both in A549 and H292 cells (Figure 2D and 2E). Colony formation assay also demonstrated the inhibition role of TIPE1 on A549 and H292 cell growth (Figure 2F and 2G). To further determined the effect of TIPE1 on lung cancer growth, siRNA targeting TIPE1 was employed to knockdown of TIPE1 in H69 cancer cells. And we found siTIPE1-2 and siTIPE1-3 significantly decreased TIPE1 expression (Figure 2H). CCK8 assay and colony formation assay demonstrated the promotion role of siTIEP1-2 and siTIPE1-3 on H69 cell growth (Figure 2I and 2J). These data demonstrated the inhibition role of TIPE1 on lung cancer cell growth and colony formation.

### TIPE1 arrests cell proliferation and induces apoptosis in lung cancer cell lines

**Figure 3:**
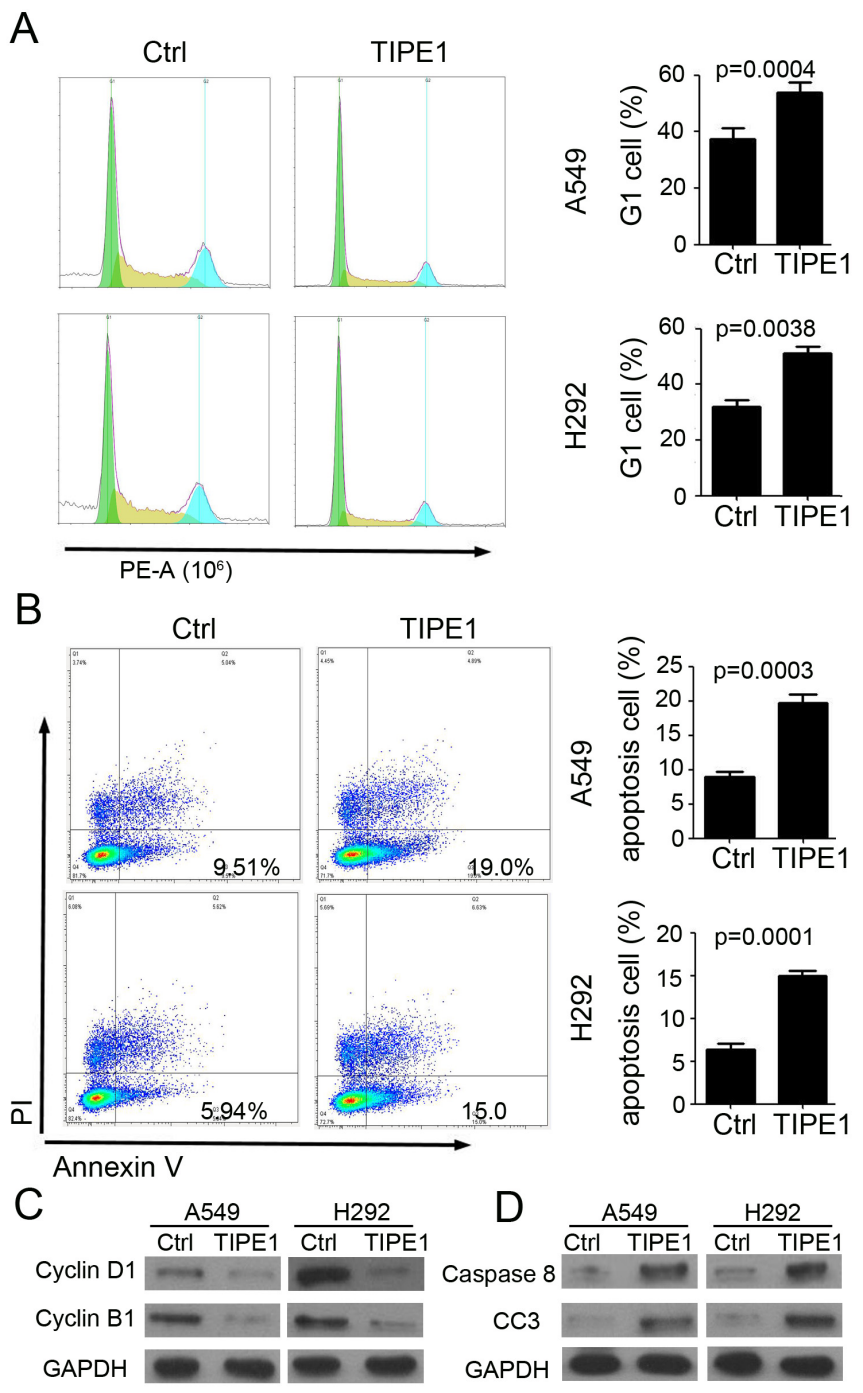
TIPE1 arrests cell proliferation and induces apoptosis in lung cancer cell lines **(A)** Assessment of cell cycle by flow cytometry in A549 and H292 cells (n=3; **, p<0.01, compared with Ctrl group). **(B)** Assessment of cell apoptosis in A549 and H292 cells by Annexin V and PI staining (n=3; **, p<0.01, compared with Ctrl group). **(C)** Western blotting was performed to detect Cyclin D1 and Cyclin B1 expression in A549 and H292 cells. GAPDH was loading as a loading control. **(D)** Western blotting was performed to detect caspase 8 and cleaved caspase 3 (CC3) expression in A549 and H292 cells. GAPDH was loading as a loading control.

Next, we investigated the mechanism of TIPE1 on lung cancer cell growth by flow cytometry. And the results indicated that TIPE1 effectively arrest A549 and H292 proliferation via inhibiting cell enter into G1 cycle (A549 cell: 17.24 % G1 cell in TIPE1 group versus 36.12% in Ctrl group; H292 cell: 17.79 % G1 cell in TIPE1 group versus 43.38% in Ctrl group; Figure 3A). Annexin V and PI staining indicated that TIPE1 induced earlier apoptosis in both A549 (Ctrl: 9.51% and TIPE1: 19.0%) and H292 (Ctrl: 5.94% and TIPE1: 15.0%) cell (Figure 3B). In addition, TIPE1 inhibited cell cycle check point protein Cyclin D1 and B1 expression (Figure 3C). Moreover, TIPE1 also promoted caspase 8 and cleaved caspase-3 expression, that are pivotal mediators involved in apoptosis (Figure 3D). The above data proved the effect of TIPE1 on lung cancer cell proliferation and apoptosis through regulating related protein expression.

### TIPE1 inhibits invasion and migration in lung cancer cells

**Figure 4:**
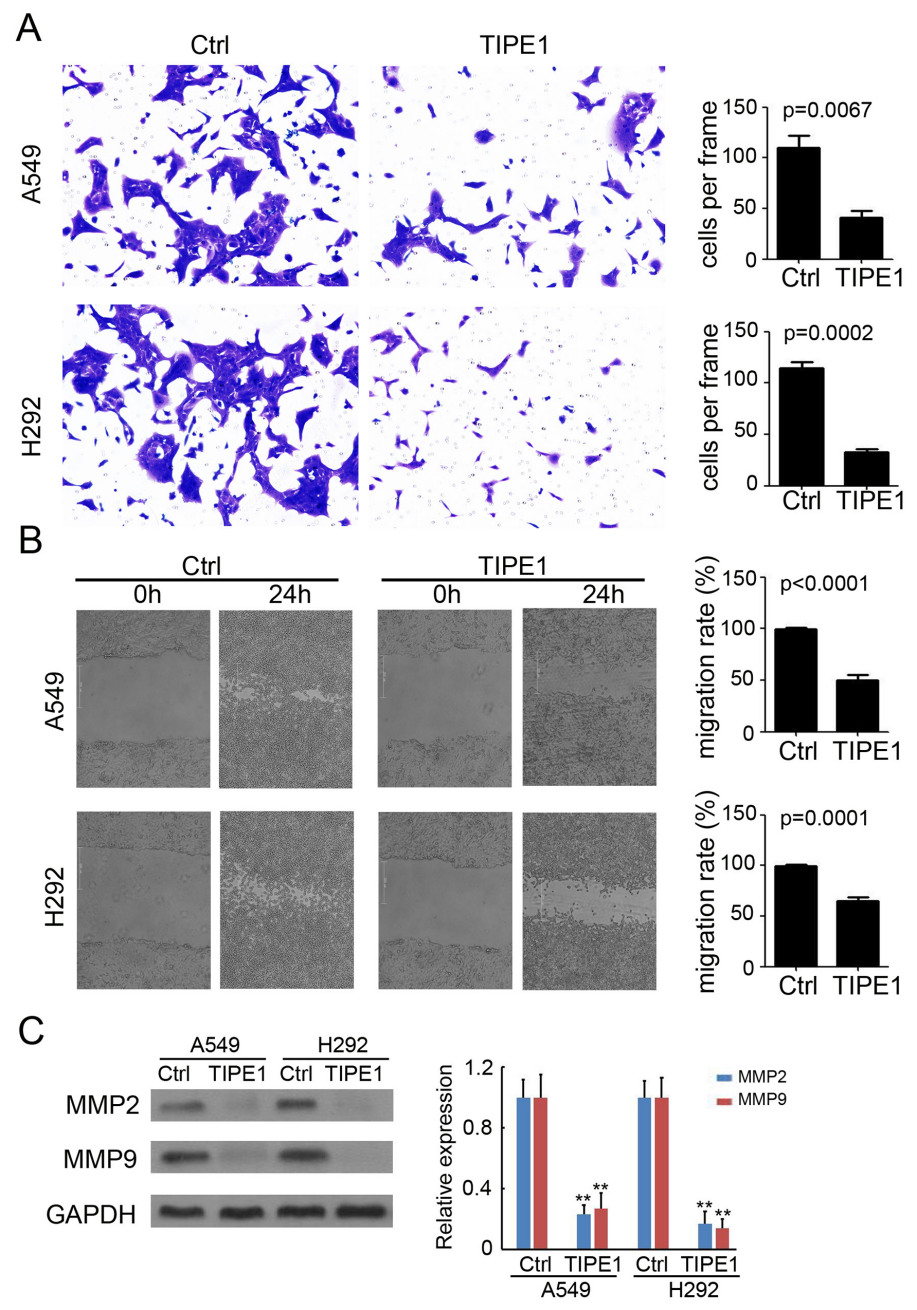
TIPE1 inhibits invasion and migration in lung cancer cells **(A)** Matrigel-based invasion assay was performed to determine the invasive properties of A549 and H292 cells (n=4; **, p<0.01, compared with Ctrl group). Scale bar=100 μm. **(B)** Wound healing assay was performed to determine the migration properties of A549 and H292 cells (n=4; **, p<0.01, compared with Ctrl group). Scale bar=200 μm. **(C)** Western blotting was performed to detect MMP2 and cleaved MMP9 expression in A549 and H292 cells. GAPDH was loading as a loading control.

An invasion assay was performed in Transwell chambers containing 8-μm pore size inserts, the upper surfaces of which were coated with Matrigel matrix. A549 and H292 cells were suspended in serum-free media and plated on the upper chambers. After 48 hours of culture, TIPE1 significantly prevented A549 and H292 cell invasion by approximately 65% and 78%, respectively (Figure 4A). Meanwhile, a wound-healing assay was used to evaluate the effect of TIPE1 on cancer cell migration. As shown in Figure 4B, compared with Ctrl group, TIPE1 dramatically blocked A549 and H292 cell migration by approximately 52% (P<0.0001) and 41% (P=0.0001), respectively. Furthermore, western blotting was performed to detect matrixmetalloproteinases (MMP) expression, which play a key role in tumor invasion and migration. We found TIPE1 significantly downregulated MMP2 and MMP9 expression by approximately 70-80% both in A549 and H292 cells (Figure 4C). Collectively, we demonstrated the inhibition role of TIPE1 on lung cancer cell invasion and migration via regulating MMP expression.

### TIPE1 inhibited tumorigenesis *in vivo*

**Figure 5:**
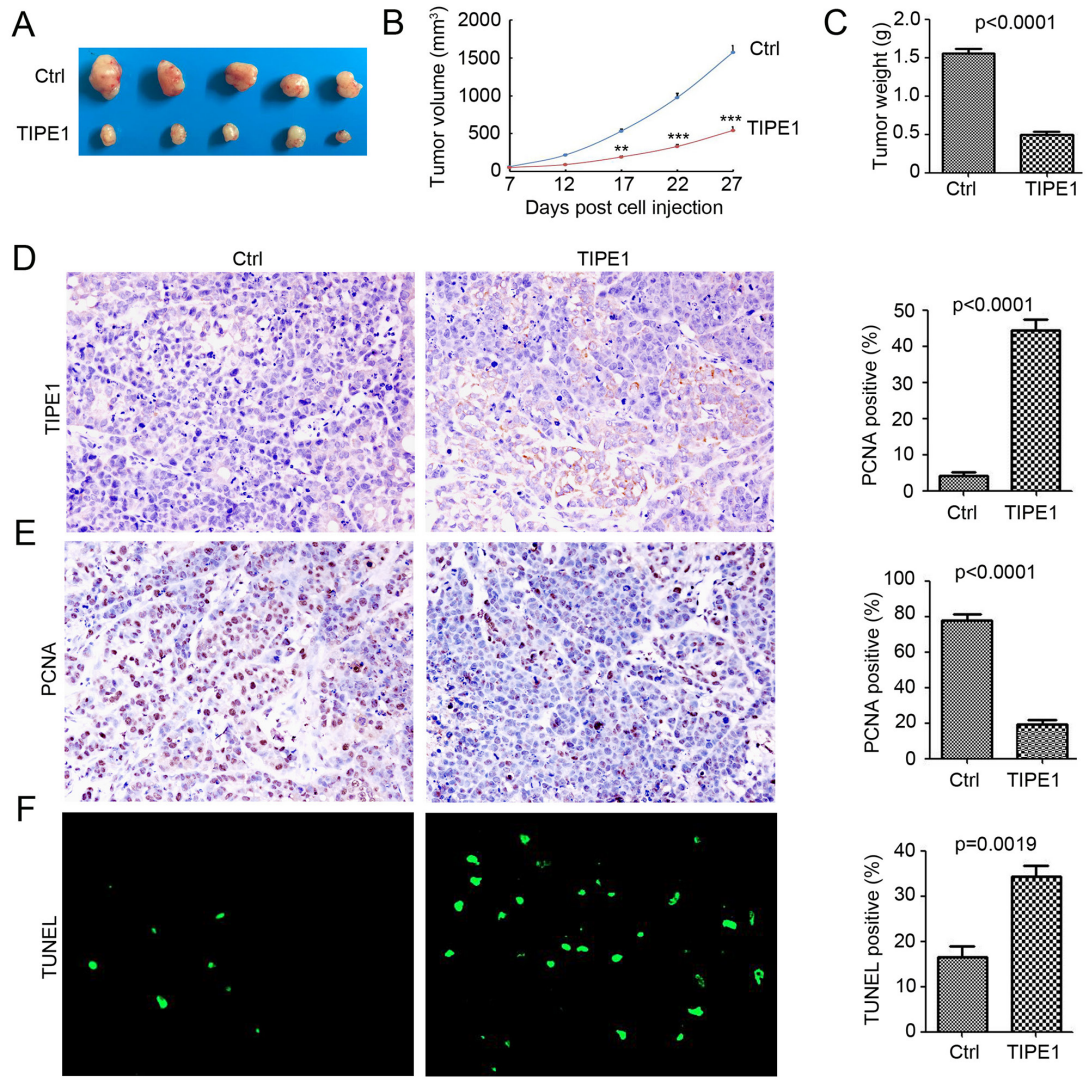
TIPE1 inhibited tumorigenesis *in vivo* **(A)** Ten BALB/c nude mice were randomly divided into two groups (5 mice per group) and subjected to injection with A549-Ctrl and A549-TIPE1 cancer cells. Representative tumors were imaged after sacrifice to visually assess tumor growth. **(B)** The average tumor volume in each group (n=5) is shown as a function of time (**, p<0.01; ***, p<0.001, compared with Ctrl group). **(C)** tumor weight in each group (n=5) is shown (**, p<0.01, compared with Ctrl group). **(D** and **E)** IHC for analysis of TIPE1 and PCNA protein expression in Ctrl and TIPE1 A549 tumor tissues. Percentage of positive cells were analyzed (n=5). **(F)** TUNEL for analysis of apoptosis cells in Ctrl and TIPE1 A549 tumor tissues. Percentage of apoptosis cells were analyzed (n=5).

Various *in vitro* results demonstrated the inhibition role of TIPE1 on tumor growth and invasion in lung cancer cells. Thus, we next investigate whether TIPE1 arrests tumor xenograft growth *in vivo*. TIPE1 A549 cells and Ctrl cells engrafted onto 10 BALB/c nude mice (5 mice per group) to monitor tumor growth. TIPE1 overexpression significantly prevented A549 lung cancer xenograft growth compared with the Ctrl control by approximately 65.8% in tumor volume (TIPE1 group: 538.27±67.21 mm^3^ versus Ctrl group: 1572.32±182.14 mm^3^; Figure 5A and 5B) and by approximately 68.4% in tumor weight (TIPE1 group: 0.49±0.04 g versus Ctrl group: 1.55±0.06 g; Figure 5C). Immunohistochemistry staining demonstrated the overexpression of TIPE1 in TIPE1 stable infected A549 cells (Figure 5D). Next, we determined the proliferation and apoptosis status by PCNA immunostaining and TUNEL assay. Less PCNA positive cells were found in the TIPE1 group (Figure 5E), whereas more TUNEL positive cells were found (Figure 5F). Collectively, TIPE1 inhibited lung cancer tumorigenesis via inhibiting proliferation and inducing apoptosis *in vivo*.

## DISCUSSION

Until now, various studied have reported the altered expression of TNFAIP8 family in the development of several types of cancers. TNFAIP8 has been demonstrated to be upregulated in gastric adenocarcinoma tissues [13], platinum-resistant epithelial ovarian cancer tissues [14], endometrial cancer tissues [15] and NSCLC tumor tissues [16]. TIPE2 expression was more pronounced in colon cancer tissues [17], but downregulated in HCC [18]. Recent study by Zhang et al have found less TIPE1 expression in HCC [12]. Cui et al have demonstrated the extensive expression of TIPE1 in variety of cells of the epithelial origin, including lung epithelial cells [19]. In our study, we found the lower TIPE1 mRNA and protein expression in NSCLC caner tissues, compared with the adjacent nontumor tissues (Figure 1). Moreover, TIPE1 mRNA and protein expression was undetectable or low level in all NSCLS cancer cell lines (Figure 2). The mechanism leading to the downregulated of TIPE1 expression in several cancer was still unclear. Epigenetic modification, such as methylation, may be the cause. It would be investigated in the further study by us.

Meanwhile, TNFAIP8 family also function as a predictor for cancer prognosis. TNFAIP8 overexpression was associated significantly with depth of invasion, lymph node metastasis and Lauren classification, and a poorer overall survival in gastric adenocarcinoma patients [13]. In TNFAIP8 upregulated endometrial cancer tissues, higher histologic grade, deeper myometrial invasion and lymph vascular space invasion and more lymph node metastasis were found, companied with poor overall survival and disease-free survival (DFS) rates [15]. Moreover, TNFAIP8 overexpression was correlated with platinum resistance in epithelial ovarian cancer and residual tumor size [14]. TIPE1 expression in HCC tissues positively correlated with tumor pathologic grades and patient survival [12]. NSCLC tumor microarray by us also demonstrated that the patients with low TIPE1 expression have dramatically shorter survival than those with high TIPE1 expression. It’s indicated that TIPE1 is a novel prognosis predictor for NSCLC patients.

TNAFIP8 family are recently identified proteins which are important for maintaining immune homeostasis. TNAFIP8 functioned as an oncogene in several cancer through promoting proliferation, metastasis and inhibiting apoptosis [13–16]. But little is known about the role of TNAFIP8 in immune [7]. TIPE2 is an essential negative regulator of inflammation and immune homeostasis. It plays anti-inflammation roles by negatively regulating T cell receptor (TCR) and Toll-like receptor (TLR) signaling [10]. According to the deficiency of TIPE1 protein in mature T or B lymphocytes [19], TIPE1 may have no function role in immune. But recently research demonstrated the induction role of TIPE1 in HCC cell apoptosis via inverses regulating Rac-1 [12].

In a Parkinson’s disease model, TIPE1 binds to FBXW5, increasing autophagy through activation of TSC2 [20]. Due to the three-dimensional structure of TIPE1 protein, TIPE1 was predicted to interact with FBXW5, caspase8 and so on [21]. In our study, we found TIEP1 arrest NSCLC cancer cell cycle, inhibited apoptosis and metastasis (Figures 3–5). Mechanism investigation found Cyclin D1, Cyclin B1, caspase 8, cleaved caspase 3, MMP2 and MMP9 were both regulated by TIPE1 in NSCLC cancer cells (Figures 3 and 4). We will put our efforts to determine the potential direct target of TIPE1 in regulating NSCLC cancer cell behaviors.

In conclusion, in the present study we showed that TIPE1 expression was dramatically decreased in the lung tumor tissues, and positively correlated with tumor patient survival. Overexpression of TIPE1 significantly diminished tumor growth and cell growth, companied with proliferation inhibition and apoptosis induction *in vivo* and *in vitro*, due to TIPE1-regulated protein expression. These findings may help to better understand the role of TIPE1 in lung cancer and provide a new candidate for prognostic indicator and molecular targeted therapies in lung cancer.

## MATERIALS AND METHODS

### Cell culture and treatment

The colorectal cancer cell lines H69, H292, Calu-1, 95-D and A549, and normal lung epithelial cell MRC-5 were cultured according to the standard protocols provided by ATCC. The lentivirus-based TIPE1 expression system was employed to infect H292 and A549 cells, and puromycin was added to select the stable TIPE1-expression cells. The lentivirus vector was used as the control.

### Tissue microarray

The cohort of 60 pairs of human malignant lung cancer tissues and their corresponding adjacent noncancerous lung tissues was purchased from Shanghai Outdo Biotech. Co. Ltd (Shanghai, China) and approved by the Ethics Committee of Taizhou Hospital. The cohort was used to determine TIPE1 expression by immunohistochemistry. Tumors and nontumors were scored by counting the number of total cells expressing the proteins as determined by TIPE1 staining, according to the previous study indicated [22]. The IHC score of TIPE1 in lung tissues were classified according to the percent of TIPE1 positive cells number in three random fields. 4, more than 25% cells are TIPE1 positive; 3, the percent cells of TIPE1 positive are between 25% and 35%; 2, the percent cells of TIPE1 positive are between 15% and 25%; 1, the percent cells of TIPE1 positive are between 10% and 20%; 0, the percent cells of TIPE1 positive are less than 5%. IHC score 2, 3 and 4 were defined as TIPE1 high expression and 0 and 1 were defined as TIPE1 low expression.

### RT-PCR

Total RNA was extracted from cells using TRIzol reagent (Invitrogen, Carlsbad, CA, USA). RNA samples (1 μg) were subjected to real-time PCR, resulting complementary DNA was analyzed in triplicate using SYBR Green (Takara, Shiga, Japan). Relative mRNA concentrations were determined by 2^-ΔΔCt^, where Ct is the mean threshold cycle difference after normalization to U6 values. The primer for TIPE1 was as follow: forward primer 5’-CAGTGACCTGCTAGATGAG-3’, reverse primer 5’-CAAGGTGCTGAGTGAAGT-3’.

### CCK8 assay

A549 and H292 cells were plated out in 100 μl of medium at a concentration of 1×10^4^ cells per well in 96-well plate. 10 μl WST-8 (Dojindo Laboratories) was added into the 96-well plate. After incubation of cells for 4 hours, the absorbance was determined at wavelength of 450 nm. The same experiments were performed in four times.

### Apoptosis assay

Apoptosis was detected using the dual staining Annexin V/PI Apoptosis Detection Kit (Keygentec, Nanjing, China) on a Cytomics FC500 flow cytometer (Beckman Coulter). The percentage of apoptotic cells in each quadrant was calculated Flow Jo Software. Each experiment was performed in triplicate.

### Western blotting

The lung cancer cells were collected and lysed on ice for 30min with the RIPA lysis buffer (Beyotime, Nanjing, China) containing 1% protease inhibitor cocktail (Sigma Aldrich). The lysis was centrifuged for 15min by 12,000 g at 4 °C, and the supernatants were collected for protein concentrations determination by the Bradford protein assay kit (Thermo Scientific, MA, USA). The protein was loaded and separated by SDS–PAGE gel electrophoresis, followed by the transfer of proteins onto the PVDF membranes (Merck Millipore). The membranes were incubated with the primary antibodies against the specific primary antibody in 5% milk TBS/T buffer overnight at 4 °C. Following incubation with horseradish peroxidase-conjugated secondary antibodies (Zsbio, Beijing, China) at room temperature for 1 h, the bands were detected using a chemiluminescent substrate ECL kit (Merck Millipore). The relative expression was determined by Image J software.

### Colony formation assay

48 hours post transfection, 1000 H292 and A549 cells were seeded into the 6 well plate, was fixed with DMEM medium containing 10% FBS. 10 days later, the plate was fixed with 4% paraformaldehyde and stained with crystal violet (Beyotime, Beijing, China). The number of colony formation were counted and analyzed.

### Cell cycle analyses

48 hours post transfection, the transfected H292 and A549 cells were collected and stained with PI for 30 mins. Then Cytomics FC500 flow cytometer (Beckman Coulter) was used to analyze the cell cycle of cells according to the fold of DNA as the previous study indicated [23]. Each experiment was performed in triplicate.

### Matrigel-based invasion assay

Matrigel (BD bioscience) was diluted (1:5) with culture medium and added into the millicell (8μm pore size). In the upper layer, 100 μl culture medium without FBS was added, whereas in the 0.5 ml culture medium with 10% FBS was added into the 24-well plate. The A549 and H292 cells (2×10^4^ cells) were added into the millicell and incubated for 48 hours. Then the cells were stained by crystal violet and photographed.

### Wound-healing assay

A549 and H292 cells were grown to 80–90% confluence in 6-well plates and a wound was made by dragging a plastic pipette tip across the cell surface afer 24 h. The phase of the wounds were recorded at 37°C for incubations of 48 h, and 3 separate experiments were performed. Image J software was used to evaluate the migration rate of 4T1 cells.

### Animal study

All of the animal studies were performed in accordance with institutional guidelines concerning animal use and care. Female BALB/c nude mice were purchased from the Model Animal Research Center of Nanjing University (Nanjing, China) and allowed to acclimate for 1 week before use. A549 cells (5×10^6^ cells per mice) were injected into the flank of female BALB/c nude mice to establish a subcutaneous cancer model. Tumor size was determined by collecting length and width measurements, and calculating the tumor volume (mm3) as (tumour length×(tumour width)^2^) ×0.52. When mice were killed (27 days after tumor cell injection), tumors from each animal were collected, weighed and used for histopathological studies.

### Immunochemistry staining

Paraffin sections were used for immunohis-tochemical analysis as previous study indicated [24]. Sections were deparaffnized in xylene, and rehydrated in PBS (pH 7.4). Antigen retrieval was done by heating for 3 min in a pressure cooker with 0.1 mol/L citrate buffer (pH6.0). After endogenous peroxide blocking and 5% normal goat serum blocking, the specific primary antibody (TIPE1, PCNA) in blocking solution were added and incubated overnight at 4 °C. The following operation was followed the instruction of immunochemistry staining kit (Zsbio, Beijing, China). The immunoreaction was visualized by using diaminobenzidine (DAB) peroxide solution and cellular nuclei were counterstained with hematoxylin. All specimens were evaluated using Olympus B×600 microscope and Spot Fiex camera.

### TUNEL assay

DeadEndTM Fluorometric TUNEL System (Promega, Madison, Wisc, USA) was performed to detect apoptotic cells in tumor tissues, following the manufacturer’s protocol. Cell nuclei with dark green fluorescent staining were defined as TUNEL-positive nuclei. Cell nuclei were counterstained with 4, 6-diamidino-2-phenylindole (DAPI, Beyotime, Beijing, China). The cells were monitored by fluorescence microscope.

### Statistical analysis

All experiments were repeated three to five times, and the data were expressed as the mean ± s.d. Statistical analysis was performed by the Student’s t-tests. Kaplan–Meier curves and the log-rank test were used to compare survival times between the groups. P<0.05 was considered statistically significant. All calculations were performed using SPSS v19.0 (SPSS Inc., Chicago, IL, USA).

## References

[1] Siegel RL, Miller KD, Jemal A (2015). Cancer statistics, 2015. CA Cancer J Clin.

[2] Ahsan H, Thomas DC (2004). Lung cancer etiology: independent and joint effects of genetics, tobacco, and arsenic. Jama.

[3] Le CH, Ko YC, Cheng LS, Lin YC, Lin HJ, Huang MS, Huang JJ, Kao EL, Wang HZ (2001). The heterogeneity in risk factors of lung cancer and the difference of histologic distribution between genders in Taiwan. Cancer Causes Control.

[4] Kerr KM, Bubendorf L, Edelman MJ, Marchetti A, Mok T, Novello S, O’Byrne K, Stahel R, Peters S, Felip E (2014). Second ESMO consensus conference on lung cancer: pathology and molecular biomarkers for non-small-cell lung cancer. Ann Oncol.

[5] Niederst MJ, Sequist LV, Poirier JT, Mermel CH, Lockerman EL, Garcia AR, Katayama R, Costa C, Ross KN, Moran T, Howe E, Fulton LE, Mulvey HE (2015). RB loss in resistant EGFR mutant lung adenocarcinomas that transform to small-cell lung cancer. Nat Commun.

[6] Tang YN, Ding WQ, Guo XJ, Yuan XW, Wang DM, Song JG (2015). Epigenetic regulation of Smad2 and Smad3 by profilin-2 promotes lung cancer growth and metastasis. Nat Commun.

[7] Lou Y, Liu S, The TIPE (2011). (TNFAIP8) family in inflammation, immunity, and cancer. Mol Immunol.

[8] Luan YY, Yao YM, Sheng ZY (2013). The tumor necrosis factor-alpha-induced protein 8 family in immune homeostasis and inflammatory cancer diseases. J Biol Regul Homeost Agents.

[9] Laliberte B, Wilson AM, Nafisi H, Mao H, Zhou YY, Daigle M, Albert PR (2010). TNFAIP8: a new effector for Galpha(i) coupling to reduce cell death and induce cell transformation. J Cell Physiol.

[10] Sun H, Gong S, Carmody RJ, Hilliard A, Li L, Sun J, Kong L, Xu L, Hilliard B, Hu S, Shen H, Yang X, Chen YH (2008). TIPE2, a negative regulator of innate and adaptive immunity that maintains immune homeostasis. Cell.

[11] Fayngerts SA, Wu J, Oxley CL, Liu X, Vourekas A, Cathopoulis T, Wang Z, Cui J, Liu S, Sun H, Lemmon MA, Zhang L, Shi Y (2014). TIPE3 is the transfer protein of lipid second messengers that promote cancer. Cancer Cell.

[12] Zhang Z, Liang X, Gao L, Ma H, Liu X, Pan Y, Yan W, Shan H, Wang Z, Chen YH, Ma C (2015). TIPE1 induces apoptosis by negatively regulating Rac1 activation in hepatocellular carcinoma cells. Oncogene.

[13] Yang M, Zhao Q, Wang X, Liu T, Yao G, Lou C, Zhang Y (2014). TNFAIP8 overexpression is associated with lymph node metastasis and poor prognosis in intestinal-type gastric adenocarcinoma. Histopathology.

[14] Liu T, Xia B, Lu Y, Xu Y, Lou G (2014). TNFAIP8 overexpression is associated with platinum resistance in epithelial ovarian cancers with optimal cytoreduction. Hum Pathol.

[15] Liu T, Gao H, Yang M, Zhao T, Liu Y, Lou G (2014). Correlation of TNFAIP8 overexpression with the proliferation, metastasis, and disease-free survival in endometrial cancer. Tumour Biol.

[16] Wang L, Song Y, Men X (2014). Variance of TNFAIP8 expression between tumor tissues and tumor-infiltrating CD4+ and CD8+ T cells in non-small cell lung cancer. Tumour Biol.

[17] Li XM, Su JR, Yan SP, Cheng ZL, Yang TT, Zhu Q (2014). A novel inflammatory regulator TIPE2 inhibits TLR4-mediated development of colon cancer via caspase-8. Cancer Biomark.

[18] Zhang YH, Yan HQ, Wang F, Wang YY, Jiang YN, Wang YN, Gao FG (2015). TIPE2 inhibits TNF-alpha-induced hepatocellular carcinoma cell metastasis via Erk1/2 downregulation and NF-kappaB activation. Int J Oncol.

[19] Cui J, Zhang G, Hao C, Wang Y, Lou Y, Zhang W, Wang J, Liu S (2011). The expression of TIPE1 in murine tissues and human cell lines. Mol Immunol.

[20] Ha JY, Kim JS, Kang YH, Bok E, Kim YS, Son JH (2014). Tnfaip8 l1/Oxi-beta binds to FBXW5, increasing autophagy through activation of TSC2 in a Parkinson’s disease model. J Neurochem.

[21] Shen P, Zhang H, Su Z, Wang S, Xu H (2015). In silico analysis of tumor necrosis factor alpha-induced protein 8-like-1 (TIPE1) protein. PLoS One.

[22] Dai L, Cui X, Zhang X, Cheng L, Liu Y, Yang Y, Fan P, Wang Q, Lin Y, Zhang J, Li C, Mao Y, Wang Q (2016). SARI inhibits angiogenesis and tumour growth of human colon cancer through directly targeting ceruloplasmin. Nat Commun.

[23] Cheng L, Yang Q, Li C, Dai L, Yang Y, Wang Q, Ding Y, Zhang J, Liu L, Zhang S, Fan P, Hu X, Xiang R (2017). DDA1, a novel oncogene, promotes lung cancer progression through regulation of cell cycle. J Cell Mol Med.

[24] Dai L, Cheng L, Zhang X, Jiang Q, Zhang S, Wang S, Li Y, Chen X, Du T, Yang Y, Tian H, Fan P, Yan N (2011). Plasmid-based STAT3-siRNA efficiently inhibits breast tumor growth and metastasis in mice. Neoplasma.

